# Development of Antimicrobial Peptides from Amphibians

**DOI:** 10.3390/antibiotics9110772

**Published:** 2020-11-04

**Authors:** Maria Luisa Mangoni, Bruno Casciaro

**Affiliations:** 1 Laboratory Affiliated to Pasteur Italia-Fondazione Cenci Bolognetti, Department of Biochemical Sciences, Sapienza University of Rome, P.le Aldo Moro 5, 00185 Rome, Italy; 2 Center For Life Nano Science@Sapienza, Istituto Italiano di Tecnologia, Viale Regina Elena 291, 00161 Rome, Italy

Since the discovery of magainins from the skin secretions of the African toad *Xenopus laevis* by Michael Zasloff in 1987, an increasing number of antimicrobial peptides (AMPs) has been identified in different anuran species and studied in detail [1,2]. As a result, frog skin is now known as one of the richest sources of natural AMPs belonging to different families [3,4]. In addition to their primary role in protecting the host from invading noxious microorganisms, they can display functions related to the host immune modulation, e.g., endotoxin neutralization, chemotaxis and wound healing activities [5,6,7]. Several studies have emphasized the potential for AMPs as new anti-infective agents with expanding properties, alone or in combination with conventional antibiotics [8,9,10,11]. Furthermore, in the rapidly evolving field of nanotechnology, several approaches have been designed to conjugate the AMPs with nanoparticulate systems to assist peptide delivery to the target sites, thus minimizing potential side effects [12,13,14,15].

This Special Issue aims to present the most recent advances in the discovery and structural-functional characterization of frog-skin AMPs for the development of novel compounds to fight the alarming concern of antibiotic resistance, and therefore for their potential usage in clinical settings. All manuscripts are full research articles with encouraging data that underline the peculiarities of AMPs as valuable alternatives to conventional drugs or as adjuvants of traditional antibiotics.

In relation to the growing number of new AMPs being discovered, synthesized and characterized, Guangshun Wang documents a bioinformatics analysis of over 1000 amphibian AMPs registered in the Antimicrobial Peptide Database in the past 18 years. The results of this analysis have shown that 99.9% of the anuran AMPs are less than 50 amino acids long, with an average length of 24 residues and a net charge of +2.5. Furthermore, it is indicated how the various amphibian peptide families (e.g., temporins, brevinins, esculentins) can be connected through multiple length-dependent therapeutic applications [16].

Ogawa and coworkers cloned 12 cDNAs encoding the biosynthetic precursors for brevinin-2SSa–2SSd, esculentin-2SSa, ranatuerin-2SSa, brevinin-1SSa–1SSd, granuliberin-SSa, and bradykinin-SSa from the skin of the wrinkled frog *Glandirana susurra* that has been classified as a new frog species endemic to Sado Island in Japan. Among these AMPs, brevinin-2SSb, ranatuerin-2SSa and granuliberin-SSa were examined for their activities against different reference strains of pathogenic microorganisms that infect animals and plants. Brevinin-2SSb was active against *Escherichia coli*, *Salmonella enterica*, *Pseudomonas aeruginosa*, *Candida albicans*, *Xanthomonas oryzae* pv. *oryzae*, *Clavibacter michiganensis* subsp. *michiganensis*, and *Pyricularia oryzae*, while ranatuerin-2SSa and granuliberin-SSa displayed antimicrobial activity against *C. albicans* and *C. michiganensis* subsp. *michiganensis* [17]. In this work, the authors also assessed the binding ability of the peptides to the bacterial endotoxins, by developing an enzyme-linked endotoxin-binding assay system, and demonstrated that both brevinin-2SSb and ranatuerin-2SSa exhibited high and moderate affinity to lipopolysaccharide and lipoteichoic acid, respectively.

The contribution of Gong and collaborators is focused on the characterization of two modified peptides derived from the cationic DRP-AC4. Amino acid substitutions were performed in order to complete the hydrophobic face of the peptide (DRP-AC4b) and increase its net charge (DRP-AC4a). This latter peptide displayed significant increased potency against bacteria than the natural counterpart, despite a notable lytic action. Furthermore, the two peptides showed a faster bactericidal rate than traditional antibiotics; this can be one of the reasons explaining why bacteria do not acquire resistance to them [18].

In the manuscript of Paduszyńska and colleagues, the antimicrobial efficacy of two lipopeptides, i.e., (C10)2-KKKK-NH2 and (C12)2-KKKK-NH2 and temporin A in combination with gentamycin were studied against bacterial biofilms formed by *Staphylococcus aureus* and *P. aeruginosa.* The peptides were able to enhance the activity of the antibiotic against staphylococcal biofilm, while no synergy in activity against planktonic cells was detected. The results of these studies should encourage further experimentation on the potential usage of AMPs as adjuvant agents in treatment of biofilm-associated infections [19]. 

Chen and coworkers contributed to this Issue with the identification of a novel brevinin-2 peptide from the skin secretion of *Sylvirana guentheri*, via cloning transcripts, and designed a series of truncated derivatives of this AMP. The derivatives exhibited significantly higher antimicrobial activity and cytotoxicity compared to the parent peptide except a Pro^14^ substituted analog, which did not fold into a helical conformation in a membrane mimicking environment. Structure–activity relationship studies indicated that position 14 plays a crucial role in the formation of the α-helix. Furthermore, structural changes at this position revealed the influence on the membrane disruption potency of the peptide on bacteria and mammalian cells [20].

In the article of Casciaro and colleagues, the antimicrobial activity of two esculentin-1 derivatives (i.e., Esc(1–18) and Esc(1–21)) was investigated against the Gram-positive *Corynebacterium jeikeium,* a commensal bacterium that colonizes human skin and that is the etiological agent of serious infections, especially in immunocompromised or hospitalized patients. Furthermore, this bacterium can be the cause of bad body smell due to the generation of volatile odorous metabolites, especially in the wet parts of the body. Esc(1–18) and Esc(1–21) showed the ability to kill this bacterium at a concentration range from 0.125–0.25 µM, along with a non-toxic profile after short- and long-term (40 min and 24 h) treatment of mammalian cells. These results reinforce the potentiality of these peptides as alternative antimicrobials against *C. jeikeium* infections and/or as additives in cosmetic products (creams, deodorants) to reduce the production of bad body odor [21].

De Santana and collaborators purified and characterized Figainin 1, a novel AMP from the cutaneous secretion of the frog *Boana raniceps.* This cationic peptide with eighteen amino acid residues is rich in leucine and isoleucine, with an amidated C-terminus and adopts an α-helix conformation in the presence of a membrane mimetic solvent, i.e., trifluoroethanol. Figainin 1 was found to be active in Gram-negative, Gram-positive bacteria and the epimastigote form of the protozoan *Trypanosoma cruzi* with a minimal concentration inhibiting microbial growth (MIC) lower than 16 μM. This AMP also showed an antiproliferative activity on cancer cells, but it was toxic on human erythrocytes. Despite its adverse effects on noncancerous cells, Figainin 1 exhibited interesting properties for the development of new anticancer agents and anti-infective drugs against pathogenic microorganisms [22].

The manuscript by Rollins-Smith’s group addresses the characterization of caerin 1, an AMP originally derived from Australian amphibians and that inhibited in vitro transmission of HIV at relatively low concentrations. Here, the authors tested the effects of caerin 1 peptides and their analogs on the viability of two species of common vaginal lactobacilli (*Lactobacillus rhamnosus* and *Lactobacillus crispatus*) in order to evaluate their safety on the vaginal microbiome. Several candidate peptides displayed limited toxicity for the lactobacilli at a concentration range that would inhibit HIV. Three of these AMPs also inhibited the growth of *Neisseria lactamica*, a closely relative species of the sexually transmissible *Neisseria gonorrhoeae* at concentrations that were significantly less harmful to the resident lactobacilli [23]. 

Finally, Barran and coworkers purified and characterized five ocellatin-related peptides from *Leptodactylus insularum* (ocellatin-1I, together with its (1–16) fragment, ocellatin-2I and its (1–16) fragment, and ocellatin) and four ocellatins from *L. nesiotus* (ocellatin-1N, -2N, -3N and -4N). Among these, ocellatin-3N showed activity against antibiotic-resistant strains of *E. coli* and *Klebsiella pneumoniae*, and reference strains of *P. aeruginosa* and *Salmonella typhimurium* (as well as *S. aureus* and *Enterococcus faecium* with an MIC of 31.25–62.5 μM). Despite the therapeutic potential of ocellatin-3N is limited by its moderate hemolytic activity, it represents a template for the design of long-acting, non-toxic, broad-spectrum antimicrobial agents to target multidrug-resistant pathogens [24].

The authors of articles published in this Issue are thanked for their contribution to the field of amphibian AMPs. We are also grateful to the Editors and to the Antibiotics Editorial Office for giving us the opportunity to edit this Special Issue and for their assistance.
